# Evaluating Surgical Approaches in Advanced Age: A Propensity-Matched Analysis of Postoperative Morbidity and Short-Term Outcomes Following Open Versus Robotic Radical Cystectomy (ASPECT Study)

**DOI:** 10.3390/cancers18142314

**Published:** 2026-07-17

**Authors:** Bara Barakat, Joerg Bauer, Mahmud Sayed, Raed Hakoub, Nico Adamini, Sameh Hijazi, Ahmed Gaafar

**Affiliations:** 1 Department of Urology, Hospital Kassel, 34125 Kassel, Germanynico.adamini@gnh.net (N.A.); 2 Faculty of Medicine, University of Duisburg-Essen, 45147 Essen, Germany; 3 Department of Urology, Hospital Ibbenbüren, 49477 Ibbenbüren, Germany; 4 Department of Urology, University Cologne, 50937 Cologne, Germany; 5 Department of Urology, Hospital Siegen, 57076 Siegen, Germany

**Keywords:** postoperative morbidity, radical cystectomy, advanced age, short-term outcomes

## Abstract

**Introduction:** The optimal surgical approach for elderly bladder cancer patients remains controversial. We compared perioperative morbidity and short-term outcomes in patients aged ≥ 75 years undergoing open radical cystectomy (ORC) versus robot-assisted radical cystectomy (RARC). **Methods:** A retrospective, multicenter cohort study was performed including 179 patients aged ≥ 75 years, of whom 101 underwent RARC between 2021 and 2025, and 78 underwent ORC between 2016 and 2020. After 1:1 propensity score matching, 138 patients were analyzed to assess perioperative complications and short-term postoperative outcomes. Pathological outcomes, including pathological stage, tumor grade, lymph node status, and positive surgical margins, were additionally compared between groups. Propensity score matching was performed based on age, body mass index (BMI), pathological stage, comorbidities, prior chemotherapy, and type of urinary diversion. **Results:** Following propensity score matching, RARC was associated with longer operative time (332 vs. 247 min; *p* < 0.001) but resulted in significantly lower blood loss (310 vs. 743 mL; *p* < 0.001), reduced transfusion rates, shorter length of hospital stay (*p* < 0.001), and fewer overall intraoperative complications (8.7% vs. 18.8%; *p* = 0.04). Patients undergoing RARC also experienced lower rates of any complications (43.5% vs. 62.3%; *p* = 0.02) and major complications (Clavien–Dindo III–V: 11.6% vs. 27.5%; *p* = 0.03). Postoperative mortality was low in both groups and did not differ significantly between RARC and ORC (1.4% vs. 2.9%; *p* = 1.00). In multivariate analysis, surgical approach independently predicted major complications, with RARC conferring a significantly lower risk (OR 0.75; 95% CI 0.51–0.88; *p* = 0.04). Analysis of the learning curve showed a significant reduction in major complications over time for RARC (OR 0.68; 95% CI 0.53–0.93; *p* = 0.01) but not for ORC. **Conclusions:** RARC offers superior perioperative outcomes, including reduced blood loss, shorter hospitalization, and lower rates of major complications, without differences in short-term pathological outcomes. These data support RARC as a safe and effective option for elderly patients undergoing radical cystectomy.

## Simple Summary

Bladder cancer is increasingly diagnosed in older patients, who often have multiple comorbidities and a higher risk of complications after surgery. Radical cystectomy is the standard curative treatment, but the optimal surgical approach in elderly patients remains debated. In this multicenter study, we compared open radical cystectomy (ORC) with robot-assisted radical cystectomy (RARC) in patients aged 75 years and older. After adjustment for baseline characteristics, RARC was associated with less blood loss, fewer blood transfusions, shorter hospital stay, and lower rates of major postoperative complications compared with ORC. Importantly, traditional risk factors such as comorbidity burden and physical status were less predictive of outcomes in the RARC group, suggesting a reduced impact of frailty with the minimally invasive approach. These findings indicate that RARC may offer a safer perioperative profile for elderly bladder cancer patients while maintaining oncological adequacy.

## 1. Introduction

Bladder cancer (BCa) is the tenth most common cancer worldwide. In 2020, there were approximately 573 thousand new cases and over 212 thousand deaths globally. It predominantly affects men, with incidence rates up to four times higher than in women [1]. The average age at which BCa is diagnosed is 73 years, which is higher than the overall average age at which cancers are diagnosed, which is 65 years [2]. The incidence of BCa increases with age; in the United States, approximately 90% of cases are diagnosed in individuals aged over 55 years, with about 80% occurring in those aged 65 and older [3]. Given the strong association between age and BCa incidence, the disease is expected to represent an increasing public health challenge in the coming decades, particularly in the context of population aging and demographic expansion [4].

According to evidence-based guidelines, radical cystectomy (RC) with urinary diversion remains the gold-standard treatment for muscle-invasive or high-risk non-muscle-invasive BCa [5,6]. RC is a complex surgical procedure and is associated with substantial postoperative morbidity, with overall complication rates reported to range from approximately 30% to 80% within 90 days after surgery [7,8]. Unfortunately, there is a paucity of evidence-based guidelines for the short- and long-term management of BCa in geriatric patients.

Patients aged ≥ 75 years frequently present with multiple comorbidities, placing them at an even higher risk of complications and mortality. In geriatric patients with bladder cancer, there is frequently a high degree of uncertainty in treatment decision-making, leading many clinicians to choose conservative or palliative approaches such as transurethral resection of the bladder tumor (TURBT) rather than curative therapies. Several studies have reported that older patients more often receive less aggressive interventions and are under-represented in curative treatment groups [9].

Traditional open radical cystectomy (ORC) has been consistently associated with prolonged recovery, higher morbidity, and increased perioperative mortality [10,11,12,13]. This disadvantage is particularly pronounced in elderly patients with significant comorbidities. In this population, 90-day mortality rates of approximately 7–11% have been reported for patients aged 75–80 years, along with a substantially increased risk of complications compared with younger cohorts. Moreover, long-term outcomes remain inferior in elderly patients, with higher one-year mortality following RC [14]. Therefore, optimizing surgical strategies in this high-risk population is of major clinical importance. The development and adoption of minimally invasive techniques, particularly robot-assisted radical cystectomy (RARC), has aimed to reduce perioperative morbidity while maintaining oncological efficacy. RARC has been associated with faster recovery, reduced hospital stays, lower morbidity, earlier return to routine activities, and preserved functional and oncological outcomes [7,15,16]. Minimally invasive approaches, particularly RARC, have been increasingly adopted worldwide. Contemporary comparative studies and meta-analyses suggest that RARC is associated with reduced blood loss, lower transfusion rates, and a shorter hospital stay compared with ORC, while providing comparable oncological outcomes in terms of lymph node yield, positive surgical margins, and intermediate-term survival [17,18,19]. Despite this increasing adoption, high-quality evidence specifically addressing outcomes in geriatric populations remains limited. Most randomized controlled trials and large comparative studies to date include relatively younger and highly selected patients, limiting the generalizability of their findings to patients aged ≥ 75 years [17,18,19]. In addition, retrospective analyses are subject to selection bias, as fitter elderly patients are more likely to be offered minimally invasive approaches, thereby confounding comparative outcome assessment [15,16]. However, the magnitude of benefit associated with RARC appears to be influenced by institutional experience, surgical volume, and perioperative care pathways such as enhanced recovery after surgery (ERAS) protocols [20,21,22].

Given the increasing life expectancy, the rising incidence of BCa, and the paucity of trials comparing RARC with ORC in geriatric patients, there is a need for controlled data on the oncological risks and benefits. The aim of this study was to evaluate the outcomes of geriatric patients undergoing RARC compared with the current standard of care, ORC.

## 2. Materials and Methods

We conducted a retrospective, observational analysis of two institutional databases including patients aged ≥ 75 years diagnosed with histologically confirmed bladder cancer who underwent RC with curative intent. Only patients with complete documentation of preoperative baseline characteristics, intraoperative parameters, pathological findings, and 30-day perioperative outcomes were eligible for inclusion. After application of exclusion criteria, a total of 179 consecutive patients were analyzed, comprising 101 patients who underwent RARC between 2021 and 2025 and 78 patients who underwent ORC between 2016 and 2020.

The study was conducted in accordance with the Declaration of Helsinki. Ethical approval was granted by the Ethics Committee of the Westphalia-Lippe Medical Association (protocol no. 2022-764-f-S; approval date: 26 January 2023). Given the retrospective nature of the analysis, the requirement for individual informed consent was waived in accordance with institutional regulations.

Preoperative assessment included standardized evaluation of patient demographics, comorbidities, and oncological disease status. Comorbidity burden was assessed using the American Society of Anesthesiologists (ASA) physical status classification system. Tumor stage was determined preoperatively based on available imaging and transurethral resection findings. All baseline variables were stratified according to surgical approach (RARC vs. ORC) and relevant clinical characteristics, including age, body mass index (BMI), ASA score, and pathological tumor stage, as summarized in Table 1.

All patients underwent radical cystectomy with pelvic lymph node dissection and urinary diversion. Urinary diversion was performed using one of three standardized techniques: ileal conduit, ureterocutaneostomy, or orthotopic neobladder. The choice of surgical approach (ORC vs. RARC) was based on a combination of institutional practice patterns, surgeon expertise, and perioperative risk assessment. In general, patients with higher surgical complexity, extensive prior abdominal surgery, or anticipated technical difficulty were more likely to undergo ORC, particularly in the earlier study period. Conversely, RARC was preferentially offered in the later study period as institutional experience with minimally invasive techniques increased. However, final treatment allocation was conducted through a multidisciplinary and patient-centered decision process, taking into account patient comorbidities, anatomical considerations, and informed patient preference. No strict randomized selection criteria were applied.

The primary outcome of interest was early oncological and perioperative endpoints, including positive surgical margin (PSM) rates and postoperative morbidity within 90 days of surgery. Postoperative complications were prospectively classified according to the Clavien–Dindo classification system and further dichotomized into minor (Clavien–Dindo grade < IIIa) and major complications (≥IIIa) for comparative analysis [23].

Secondary outcomes included operative and recovery-related parameters. Intraoperative variables comprised operative time, estimated blood loss (EBL), and perioperative blood transfusion rates. Postoperative recovery metrics included time to first oral intake, time to bowel function recovery (exsufflation), time to catheter removal, length of hospital stay, and intensive care unit (ICU) stay. Oncological outcomes included pathological tumor stage and grade, which were assessed according to the 2004 TNM classification system and the World Health Organization (WHO) grading system, respectively [24].

Patients with a history of pelvic radiotherapy or those undergoing partial bladder resection were excluded to ensure a homogeneous study cohort. Additionally, patients with incomplete medical records or insufficient perioperative follow-up were excluded (n = 11). Due to the retrospective design of the study, certain variables were not available for all patients; therefore, subgroup analyses were performed on datasets with complete information where applicable.

To reduce selection bias and improve comparability between the two surgical groups, propensity score matching was performed based on relevant preoperative covariates (including age, BMI, ASA score, and tumor stage), resulting in balanced cohorts for comparative outcome analysis.

## 3. Statistical Data Evaluation

Data were independently reviewed for internal consistency by two investigators. Baseline characteristics of patients undergoing ORC and RARC were first compared. Categorical variables were analyzed using Pearson’s chi-squared test, and continuous variables were compared using the Mann–Whitney U test. Continuous variables (e.g., age, BMI) are presented as medians with interquartile ranges (IQRs), and categorical variables (e.g., gender, ASA score, Charlson comorbidity index, comorbidities, operative time, pathological T stage, lymph node dissection, positive surgical margins, type of urinary diversion, and complications) are reported as frequencies and percentages.

To account for potential selection bias, observed differences in baseline characteristics between ORC and RARC patients were addressed using propensity score (PS) matching. A logistic regression PS model was constructed for surgical approach (ORC vs. RARC), including age, BMI, ASA score, prior surgery or radiotherapy, and clinical T and N stage. Given inherent baseline differences between groups, a 1:1 PS-matched analysis was performed to adjust for these variables. Propensity score matching was used to mitigate selection bias inherent to conventional multivariable adjustment approaches.

Finally, logistic regression analyses were used to assess associations between variables, and univariate and multivariate Cox proportional hazards models with a stepwise approach were applied to identify risk factors for overall mortality across surgical groups. Two-sided *p*-values < 0.05 were considered statistically significant. All analyses were performed using SPSS v.22.0 (IBM Corp., Armonk, NY, USA) and Stata-SE 15 (StataCorp LP, College Station, TX, USA).

## 4. Results

### 4.1. Baseline Characteristics

A total of 179 patients aged 75 years or over with complete perioperative data were included in the study. Of these, 78 underwent ORC (median age 78.1 years, IQR 75–88) and 101 underwent RARC (median age 79.4 years, IQR 75–87). Baseline characteristics and perioperative outcomes, stratified by surgical approach, are detailed in Table 1.

The overall burden of severe comorbidity was modest, with only a minority of patients presenting with a Charlson comorbidity index (CCI) of at least 3 or ASA score of at least 3. The most prevalent comorbid conditions were coronary artery disease (n = 138; 77.1%), hypertension (n = 121; 67.6%), diabetes mellitus (n = 70; 39.1%), chronic kidney disease (n = 36; 20.1%) and chronic obstructive pulmonary disease (n = 31; 17.3%). Notably, over half of the patients (n = 98; 54.7%) required perioperative bridging of chronic anticoagulation, underscoring the clinical complexity of this population. The proportion of patients with prior abdominal surgery and/or chemotherapy was comparable between groups before matching (31.7–28.2%; *p* = 0.74) and identical after matching (31.9% in each group). Prior to matching, the RARC group had a significantly higher proportion of ASA Class III patients (65.3% vs. 34.6%; *p* < 0.05); propensity score matching achieved balance in ASA class distribution between groups.

### 4.2. Primary Outcomes

With respect to perioperative outcomes, significant differences between groups were observed for operative time and estimated blood loss (EBL). The RARC cohort had a longer operative time than the ORC cohort (332 vs. 247 min; *p* < 0.001). Conversely, EBL was significantly lower in the RARC cohort (median 310 mL vs. 743 mL in ORC; *p* < 0.001). In line with this, perioperative transfusions were administered more frequently in the ORC group.

No association was found between oral anticoagulation and complication severity (χ^2^ test, *p* = 0.634). Postoperatively, both serum albumin and hemoglobin levels declined significantly in both groups, with a greater reduction in hemoglobin observed following RARC.

Length of hospital stay was significantly shorter in the RARC group (*p* < 0.001). No between-group differences were detected regarding urinary diversion, pathological outcomes, and PSM. Preoperative characteristics of the overall cohort are summarized in Table 2.

### 4.3. Secondary Outcomes

Intraoperative complications occurred less frequently in the RARC group than in the ORC group (8.7% vs. 18.8%; *p* = 0.04). Likewise, the overall rate of postoperative complications within 90 days of any Clavien–Dindo classification (CDC) grade was lower following RARC (43.5% vs. 62.3%; *p* = 0.02) (Table 3).

**Figure 1:**
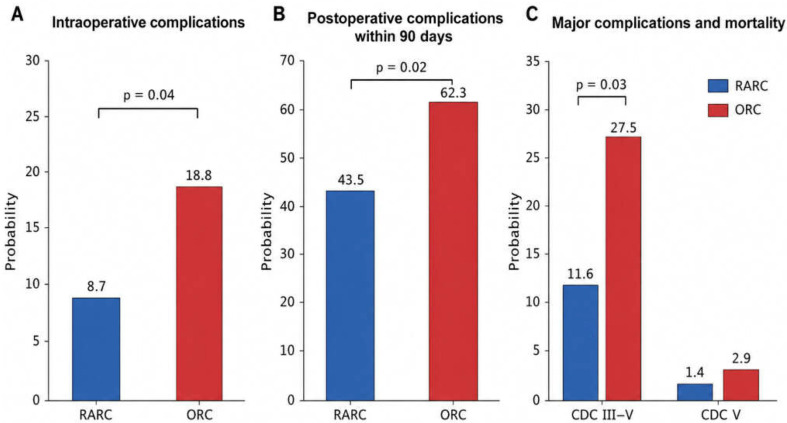
Propensity score-matched complication analysis. *ORC*: open radical cystectomy; *RARC*: robot-assisted radical cystectomy; *CDC*: Clavien–Dindo classification.

**Figure 2:**
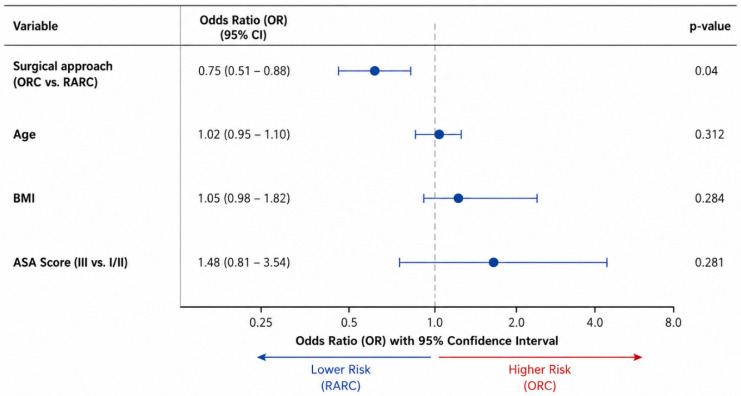
Predictors of postoperative complications within 90 days: multivariable logistic regression analysis. *ORC*: open radical cystectomy; *RARC*: robot-assisted radical cystectomy; *BMI*: body mass index; *ASA*: American Society of Anesthesiologists.

A significant reduction in major complications (Clavien–Dindo III–V) within 90 days was observed with RARC compared to ORC for the primary endpoint (11.6% vs. 27.5%; *p* = 0.03) (Figure 1). Postoperative mortality (Clavien–Dindo V) was low in both groups and did not differ significantly between RARC and ORC (1.4% vs. 2.9%; *p* = 1.00). The most frequent complications in both groups were wound dehiscence, infectious events and postoperative ileus (Table 4). Univariate and multivariate logistic regression analyses identified surgical approach as an independent predictor of major complications within 90 days (*p* < 0.001), whereas sex, BMI, ASA score, operative time, CCI, and tumor grade showed no significant association. In multivariate analysis, surgical approach independently predicted major complications, with RARC conferring a significantly lower risk (OR 0.75; 95% CI 0.51–0.88; *p* = 0.04) (Figure 2). No significant differences in morbidity or mortality were observed between continent and incontinent urinary diversions.

### 4.4. Sensitivity Analyses

Supplementary sensitivity analyses were performed to assess the robustness of the primary endpoints and to explore potential sources of bias. To evaluate the potential influence of the learning curve on postoperative complications, we compared the incidence of CD grade III or higher between the years 2016–2020 and 2021–2025. No significant difference was observed in the ORC cohort (OR 1.77, 95% CI 1.12–1.95; *p* = 0.44), whereas a significant reduction was noted in the RARC cohort (OR 0.68, 95% CI 0.53–0.93; *p* = 0.01).

## 5. Discussion

With ongoing population aging, BCa incidence in older adults will continue to increase, directly amplifying the clinical and economic burden on healthcare systems [15]. RC remains the standard curative treatment for muscle-invasive and selected high-risk non-muscle-invasive disease; however, elderly patients represent a particularly high-risk population due to reduced physiological reserve, multimorbidity, and increased vulnerability to perioperative stress. Consequently, refining surgical strategies for geriatric patients has become a central issue in contemporary uro-oncological practice [14,15,20].

### 5.1. Comorbidity and BMI as Predictors of Postoperative Risk

Comorbidity burden, commonly quantified using the Charlson comorbidity index (CCI), is a consistently validated predictor of postoperative morbidity and mortality following radical cystectomy. Prior studies have demonstrated a strong association between increasing CCI and both short-term complications and reduced survival outcomes [21,22]. In addition, ASA status reflects global physiological fitness and has been shown to independently correlate with perioperative outcomes in elderly cystectomy cohorts. Our findings confirm and extend this evidence by demonstrating that both CCI and ASA are strong predictors of complications after ORC, but lose their discriminative power in the RARC cohort, suggesting that surgical approach modifies the prognostic relevance of baseline frailty.

This effect is likely mediated by reduced surgical trauma associated with the robotic approach, including smaller incisions, decreased intraoperative blood loss, and a blunted systemic inflammatory and stress response. In elderly patients with limited cardiopulmonary reserve, even moderate perioperative stress can trigger a cascade of complications such as delayed mobilization, infection, or cardiopulmonary decompensation. Thus, RARC may function as a procedural “risk modifier”, partially decoupling baseline comorbidity from postoperative outcomes and thereby mitigating the clinical impact of frailty in high-risk patients.

These findings should be interpreted in the context of existing evidence on established perioperative risk factors in radical cystectomy. Using a standardized methodology, Roghmann et al. further demonstrated that both CCI and BMI are significant risk factors for major complications [13]. Consistent with prior reports linking elevated BMI to wound-related complications, including surgical site infection and wound dehiscence [15,25], our findings confirm BMI as an independent predictor of these complications in patients undergoing ORC.

Importantly, our comparative analysis of RARC and ORC in patients aged ≥ 75 years reveals a differential impact of established risk factors between surgical approaches. While higher comorbidity burden (CCI ≥ 3) and impaired physical status (ASA ≥ 3) were significantly associated with both overall and major complications in the ORC cohort, these associations were not observed in patients undergoing RARC. Despite the higher prevalence of comorbidities in geriatric patients, RARC was associated with a significant attenuation of postoperative risk compared with ORC. Collectively, these findings suggest that although comorbidity burden remains a critical determinant of surgical risk in elderly patients, its impact may be mitigated by minimally invasive approaches such as RARC.

### 5.2. Complication Profile

The complication rate following RC has been the focus of several retrospective series [7,26,27,28,29,30,31]. Consistent with these reports, the overall rate of high-grade complications was significantly higher for ORC compared with RARC, although the absolute difference narrowed in subanalyses restricted to high-grade events. In our elderly cohort, RARC was associated with a significantly lower rate of high-grade complications than ORC, suggesting relative advantages in postoperative rehabilitation. This benefit extended across the distribution of complication grades and 30-day mortality, indicating a clinically meaningful reduction in early postoperative risk for older patients. Several limitations of this study should be acknowledged. Firstly, the retrospective design relied on follow-up to obtain survival data, which may have limited the accuracy of information regarding the precise timing of recurrence and death. Secondly, the sample size was relatively small, so it is possible that some patients over 75 years old, particularly those in poorer physical condition, were not considered candidates for such a major procedure. Consequently, our findings may primarily reflect the outcomes of older patients with relatively preserved functional status and a more favorable baseline health profile. Thirdly, the retrospective nature of the study precluded an assessment of postoperative quality of life. Future prospective, randomized studies incorporating age, comorbidity burden and urinary diversion stratification, along with long-term follow-up, are needed to evaluate the impact of RARC on elderly patients more definitively.

### 5.3. Comparative Evidence on RARC in Elderly and High-Risk Populations

These findings are consistent with recent population-based and multicenter analyses demonstrating improved perioperative outcomes with RARC compared to ORC in elderly and octogenarian patients [32,33,34,35,36,37,38]. Large database studies have reported reduced transfusion rates, shorter hospital stay, and lower early mortality following RARC, although the magnitude of benefit appears attenuated in very elderly subgroups [11,12]. Similarly, propensity-matched analyses have confirmed that age alone does not independently predict high-grade complications after RARC when adjusting for baseline comorbidity and functional status [3,7]. Importantly, recent evidence suggests that even patients aged ≥ 85 years may safely undergo RARC in high-volume centers, with comparable oncological outcomes and acceptable perioperative morbidity, further supporting a role for minimally invasive surgery in carefully selected frail patients [10,39,40]. Comparable propensity score-matched methodology has also been applied to assess perioperative morbidity in relation to neoadjuvant chemotherapy status prior to radical cystectomy, with matched analyses showing that NAC exposure was not associated with a higher risk of high-grade complications, further supporting the utility of PSM designs in disentangling true complication risk factors from confounding by indication [41].

A relevant limitation of this study is the temporal separation of the treatment cohorts. ORC patients were treated between 2016 and 2020, whereas RARC patients were treated between 2021 and 2025. This introduces a potential period effect, which may act as an important source of confounding. During this time, several aspects of perioperative care likely evolved, including the implementation and refinement of enhanced recovery after surgery (ERAS) protocols, improvements in anesthetic and perioperative management, and increasing institutional and surgeon experience with both open and robotic techniques.

Although propensity score matching was used to balance baseline patient and tumor characteristics, residual confounding due to unmeasured time-dependent factors cannot be excluded. Therefore, part of the observed improvement in perioperative outcomes in the RARC cohort may reflect general advancements in perioperative care over time rather than the surgical approach alone.

Consequently, these findings should be interpreted as reflecting a combination of surgical technique and temporal improvements in perioperative management. Future prospective, ideally contemporaneous comparative studies are required to more precisely isolate the independent effect of RARC versus ORC. Another limitation is the restricted assessment of oncological outcomes. Due to the relatively recent implementation of RARC and the shorter follow-up duration of this cohort, long-term oncological endpoints such as recurrence-free survival, cancer-specific survival, and overall survival could not be reliably assessed. Therefore, our findings primarily address perioperative safety and short-term outcomes rather than definitive oncological equivalence between surgical approaches.

## 6. Conclusions

In this retrospective analysis, RARC was associated with a significantly lower overall complication rate compared with ORC in elderly patients. The robotic approach conferred marked reductions in intraoperative blood loss and length of hospital stay, reflecting a more favorable perioperative profile. Furthermore, higher comorbidity burden (CCI ≥ 3) and impaired physical status (ASA ≥ 3) retained predictive value for postoperative complications and short-term survival in the ORC cohort, but not in RARC patients. Overall, these findings indicate that RARC is a safe and effective surgical strategy for muscle-invasive or high-risk non-muscle-invasive BCa in geriatric patients, offering reduced intra- and postoperative risk and accelerated recovery relative to ORC.

## Figures and Tables

**Table 1:** Baseline characteristics and clinical characteristics of all included patients before and after propensity score matching.

Variable	Before Propensity Matching				After Propensity Matching			
	ORC (n = 78)	RARC (n = 101)	SMD	*p*-Value	ORC (n = 69)	RARC (n = 69)	SMD	*p*-Value
**Gender, n (%)**			−0.08	0.70			−0.04	0.61
**Male**	60 (76.9)	75 (74.2)			50 (72.6)	52 (75.4)		
**Female**	18 (23.1)	23 (25.7)			19 (27.5)	17 (24.6)		
**Age, median (IQR), years**	78.1 (75–88)	79.4 (75–87)	0.08	0.14	78.8 (75–87)	79.0 (75–87)	0.06	0.58
**BMI, median (IQR)**	26.7 (23–33)	26.9 (24–33)	0.22	0.58	26.5 (24–31)	26.5 (24–31)	0.01	0.87
**ASA score, n (%)**			0.92	<0.001			0.03	0.84
**I**	12 (15.4)	15 (14.8)			4 (5.8)	4 (5.8)		
**II**	39 (50.0)	20 (19.8)			30 (43.5)	30 (43.5)		
**III**	27 (34.6)	66 (65.3)			35 (50.7)	35 (50.7)		
**CCI [no. (%)]**			0.84	0.15			0.02	0.81
**3**	32 (41)	16 (15.8)			13 (18.8)	5 (7.2)		
**4**	37 (47.4)	20 (19.8)			27 (39.1)	25 (36.2)		
**>5**	9 (11.5)	65 (64.3)			29 (42.0)	39 (56.5)		
**Prior surgery or chemotherapy ^a^, n (%)**	22 (28.2)	32 (31.7)	0.03	0.77	22 (31.9)	22 (31.9)	0.00	1.00
**Clinical T stage, n (%)**			0.26	0.06			0.02	0.78
**cT1 + CIS**	39 (50)	28 (27.7)			26 (37.7)	26 (37.7)		
**≥cT2**	39 (50)	73 (72.3)			43 (62.3)	43 (62.3)		
**pN+**	7 (9)	11 (10.9)			5 (7.2)	5 (7.2)	0.00	1.00

^a^ Status following prior abdominal surgery and/or pelvic radiation therapy. *Abbreviations*: *SMD*: standardized mean difference; *IQR*: interquartile range; *ORC*: open radical cystectomy; *RARC*: robot-assisted radical cystectomy; *CCI*: Charlson comorbidity index; *ASA*: American Society of Anesthesiologists; *BMI*: body mass index (calculated as weight in kilograms divided by height in meters squared). Percentages have been rounded and may not add up to 100.

**Table 2:** Intra- and postoperative outcomes and type of urinary diversion in the matched cohort (n = 138).

Variable After Propensity Matching	ORC (n = 69)	RARC (n = 69)	*p*-Value
**OP time [min], median (IQR)**	247 (181–297)	332 (302–389)	<0.001
**EBL [mL], median (IQR)**	743 (435–987)	310 (224–455)	<0.001
**Anticoagulants [no. (%)]**			0.87
**No**	41 (59.4)	39 (56.5)	
**Yes**	28 (40.6)	30 (43.5)	
**Length of stay [days], median (IQR)**	18 (15–24)	12 (9–17)	<0.001
**Positive surgical margins [no. (%)]**			0.67
**R0**	60 (87)	59 (85.5)	
**R1**	9 (13)	10 (14.5)	
**LNI [no. (%)]**	5 (7.2)	5 (7.2)	1.0
**Urinary diversion [no. (%)]**			0.33
**Ileal conduit**	49 (71)	55 (79.7)	
**Neobladder**	5 (7.2)	7 (10.1)	
**Ureterostomy**	15 (21.7)	7 (10.1)	

*Abbreviations*: *IQR*: interquartile ranges; *ORC:* open radical cystectomy; *RARC*: robot-assisted radical cystectomy; *EBL*: estimated blood loss; *LNI*: lymph node invasion. Percentages have been rounded and may not add up to 100.

**Table 3:** Postoperative outcomes: frequency of minor and major complications in the matched cohort (n = 138).

Variable After Propensity Matching	ORC (n = 69)	RARC (n = 69)	*p*-Value
**Intraoperative complications [no. (%)]**			0.04
**no**	56 (81.2)	63 (91.3)	
**yes**	13 (18.8)	6 (8.7)	
**Surg. complications (CDC) within 90 days [no. %)]**			0.008
**no**	26 (37.7)	39 (56.5)	
**I**	5 (7.2)	6 (8.7)	
**II**	19 (27.5)	16 (23.2)	
**IIIa**	8 (11.5)	4 (5.8)	
**IIIb**	7 (10.1)	2 (2.9)	
**IV**	2 (2.9)	1 (1.4)	
**V**	2 (2.9)	1 (1.4)	
**Complications within 90 days**			0.02
**infection**	12 (28)	13 (43.3)	
**ileus**	10 (23.3)	6 (20)	
**wound dehiscence**	8 (18.6)	7 (23.3)	
**bleeding**	4 (9.3)	1 (3.3)	
thromboembolic events	3 (7)	1 (3.3)	
cardiopulmonary complications	6 (13.9)	2 (6.6)	
**Total complications, n (%)**	43 (62.3)	30 (43.5)	0.02
**Major complications** **(III–V), n (%)**	19 (27.5)	8 (11.6)	0.03

*Abbreviations*: *Pts*: patients; *IQR*: interquartile range; *CDC*: surg. complications according to Clavien–Dindo classification. Percentages have been rounded and may not add up to 100.

**Table 4:** Odds ratios (ORs) with a 95% confidence interval (CI) and *p*-values for uni- and multivariate logistic regression of postoperative complications within 90 d.

Dependent: Complications Within 90 D	Univariate OR (95% CI)	*p*-Value	Multivariate OR (95% CI)	*p*-Value
**Surgical approaches [no. (%)]**				
**ORC**				
**RARC**	1.81 (0.67–0.89)	0.05	0.75 (0.51–0.88)	0.04
**Age [no. (%)]**	2.19 (0.89–5.53)	0.093	1.02 (0.95–1.10)	0.312
**BMI**	1.89 (0.91–3.92)	0.321	1.05 (0.98–1.82)	0.284
**ASA score (III vs. I/II)**	1.77 (0.71–5.46)	0.421	1.48 (0.81–3.54)	0.281

*Abbreviations*: *ASA*: American Society of Anesthesiologists; *BMI*: body mass index.

## Data Availability

No new data were created or analyzed in this study. Data sharing is not applicable to this article.

## Abbreviations

Bladder cancer (*BCa*); radical cystectomy (*RC*); open radical cystectomy (*ORC*); robot-assisted radical cystectomy (*RARC*); American Society of Anesthesiologists (*ASA*); Clavien–Dindo classification (*CDC*); estimated blood loss (*EBL*); interquartile ranges (*IQRs*); body mass index (*BMI*); Charlson comorbidity index (*CCI*); positive surgical margin (*PSM*).
